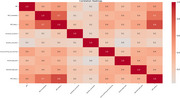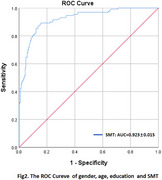# The Role of the Spatial Memory Test in Early Detection of Mild Cognitive Impairment: Insights from the Shanghai Brain Aging Study

**DOI:** 10.1002/alz70857_101483

**Published:** 2025-12-25

**Authors:** Wenkui Xiong, Zhen Gu, Bo Hong, Ling Yue, Shifu Xiao, Jian Wang, Weiyun Jiang, Naiyan Zeng

**Affiliations:** ^1^ Shanghai Mental Health Center, Shanghai Jiao Tong University School of Medicine, Shanghai, China; ^2^ Shanghai Jiao Tong University School of Medicine, Shanghai, Shanghai, China; ^3^ Yu Kang Biotechnology Co., LTD, jiaxing, Zhejiang, China

## Abstract

**Background:**

Mild cognitive impairment (MCI) is an intermediate state between normal aging and dementia, essential for early detection of dementia. The Spatial Memory Test (SMT), a sensitive digital assessment of visuospatial ability which based on the Four Mountains Test (4MT) technology, may serve as a prodromal indicator of cognitive impairment. This study aims to analyze the possibility of using the SMT to assist in the diagnosis of MCI from a community‐based cohort.

**Method:**

Samples were obtained from the Shanghai Brain Aging Study and divided into Normal Cognition (NC) and MCI groups. We compared differences in SMT scores between these cognitive diagnosis groups and evaluated the diagnostic efficacy based on regression analysis of SMT scores.

**Result:**

A total of 336 elderly individuals were included, comprising 206 in the NC group and 130 in the MCI group. The NC group exhibited higher SMT scores than the MCI group (9.95±1.74 vs 6.38±1.93, *p* <0.001). Additionally, we analyzed the correlation between the SMT and a set of neuropsychological tests (NTB). The SMT was significantly associated with AVLT‐immediately, AVLT‐delay, functional connection, visual matching and reasoning, and forward and backward Digit Span (*p* <0.001). Furthermore, after including gender, age, and education, the SMT demonstrated excellent ability to predict MCI (AUC = 0.923, *p* <0.001).

**Conclusion:**

The study showed that SMT had a good ability in detection of MCI and could reflect multiple cognitive function domains. Therefore, to establish a SMT‐based diagnostic model may provide a potential viable option for identifying cognitive impairment and may be especially useful in screening community‐dwelling elderly for early detection of dementia.